# Functional Amino Acids and Autophagy: Diverse Signal Transduction and Application

**DOI:** 10.3390/ijms222111427

**Published:** 2021-10-22

**Authors:** Chunchen Liu, Linbao Ji, Jinhua Hu, Ying Zhao, Lee J. Johnston, Xiujun Zhang, Xi Ma

**Affiliations:** 1 State Key Laboratory of Animal Nutrition, College of Animal Science and Technology, China Agricultural University, No. 2, Yuanmingyuan West Road, Haidian District, Beijing 100193, China; liuchunchenlcc@163.com (C.L.); jilinbao0126@cau.edu.cn (L.J.); hujinhua@cau.edu.cn (J.H.); sy20193040684@cau.edu.cn (Y.Z.); 2 West Central Research & Outreach Center, University of Minnesota, Morris, MN 56267, USA; johnstlj@umn.edu; 3 College of Public Health, North China University of Science and Technology, Qinhuangdao 063210, China

**Keywords:** autophagy, autophagy-related diseases, functional amino acids, mTORC1, signal transduction

## Abstract

Functional amino acids provide great potential for treating autophagy-related diseases by regulating autophagy. The purpose of the autophagy process is to remove unwanted cellular contents and to recycle nutrients, which is controlled by many factors. Disordered autophagy has been reported to be associated with various diseases, such as cancer, neurodegeneration, aging, and obesity. Autophagy cannot be directly controlled and dynamic amino acid levels are sufficient to regulate autophagy. To date, arginine, leucine, glutamine, and methionine are widely reported functional amino acids that regulate autophagy. As a signal relay station, mammalian target of rapamycin complex 1 (mTORC1) turns various amino acid signals into autophagy signaling pathways for functional amino acids. Deficiency or supplementation of functional amino acids can immediately regulate autophagy and is associated with autophagy-related disease. This review summarizes the mechanisms currently involved in autophagy and amino acid sensing, diverse signal transduction among functional amino acids and autophagy, and the therapeutic appeal of amino acids to autophagy-related diseases. We aim to provide a comprehensive overview of the mechanisms of amino acid regulation of autophagy and the role of functional amino acids in clinical autophagy-related diseases and to further convert these mechanisms into feasible therapeutic applications.

## 1. Introduction

A delicate balance of organelle generation and renovation is required to guarantee normal differentiation and development in mammalian cells. To maintain the intracellular metabolic balance, cells have evolved a self-degradative process, called autophagy. The term “autophagy” was coined when lysosomes were discovered [1]. Autophagy is also a housekeeping process that delivers unwanted materials of endogenous or exogenous origin through the cytosol into the lysosome for breakdown. In particular, autophagy occurs when cells face invading microbes, stress from nutrient withdrawal, dysfunctional organelles, and pathogenic proteins. Dysregulated autophagy links exist in numerous pathologies, especially cancer, neurodegeneration, aging, obesity and a range of complications, including inflammatory disorders, cardiovascular disease, and diabetes [2]. Considerable passion for research on the regulation of autophagy has emerged, which may lead to therapeutic options in clinical settings [3]. Regulating autophagy directly to treat disease is impractical; therefore, the research has changed to focus on other indirect methods.

In most conditions, autophagy can play a physiological role in balancing the body’s amino acid pools, and numerous amino acids (AA) are critical regulatory signals for autophagy in turn [4]. Generally, in response to a certain degree of the amino acid starvation, autophagy degradation promotes the release of AA to protect cells. Additionally, autophagy activation maintains the intracellular amino acid pool [5]. Under the stress of nutrient deficiency or excess, amino acids can regulate autophagy via signal transduction [6]. Amino acid regulation of autophagy is a burgeoning field of investigation. In particular, functional amino acids (FAAs) (e.g., arginine, leucine, glutamine, and methionine) participate in protein synthesis and homeostasis, which lead to cell signaling transduction by certain protein kinases [7]. Mammalian target of rapamycin complex 1 (mTORC1), a signal transduction regulator, links FAAs with autophagy; thus, FAAs may hold great promise for the prevention and treatment of autophagy-related diseases [7].

In this review, after introducing our understanding of autophagy and the amino acid sensing mechanism briefly, this review describes the specific autophagy regulated by signal transduction of functional amino acids (e.g., arginine, leucine, glutamine, and methionine) and the association between autophagy-related diseases and functional amino acids. We provide an overview of the impacts of amino acids with autophagy and propose the novel clinical treatment of autophagy-related diseases based on the indirect regulation of functional amino acids.

## 2. Mechanisms of Autophagy Induction

**Figure 1:**
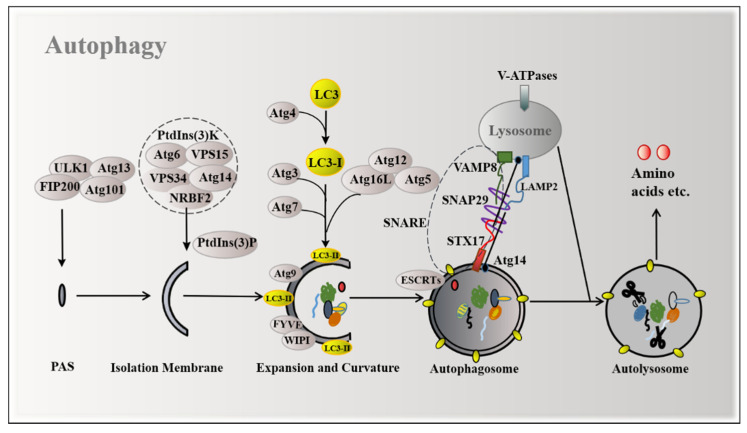
Mechanisms of autophagy induction. The ULK1 complex, PtdIns(3)K complex, and two ubiquitination systems are involved in the formation of autophagosomes. Atg14 promotes the formation of STX17-SNAP29-VAMP8-reconstituted proteoliposomes to form autolysosomes.

Improper nutrient status is the most common cause of autophagy. AMP-activated protein kinase (AMPK) and mTORC1 are major nutrient sensors that are used to monitor the energy source and amino acid pool, respectively. A lack of energy leads to an increased AMP/ATP ratio, activating AMPK via phosphorylation to inhibit mTORC1 [8], thereby promoting the induction of autophagy (Figure 1).

Autophagy is a dynamic process of removal and renovation in cells to maintain homeostasis. Accordingly, disorder of this process leads to many human diseases. Extensive research has shown that autophagy can be divided into microautophagy, macroautophagy, and chaperon-mediated autophagy (CMA) in eukaryotic cells [9]. The best studied of these is macroautophagy, which maintains the main catabolic mechanism and organelle quality control. In this review, we mainly focus on macroautophagy and refer to macroautophagy vesicles as autophagosomes. Briefly, the biogenesis of autophagosomes originates from the assembly of pre-autophagosome structures (PAS), whereby the cargo (e.g., cytoplasmic organelles, proteins, and macromolecules) in the cytoplasm is first surrounded by “isolation membrane” from the endoplasmic reticulum and the Golgi apparatus, which expands outward and eventually closes, forming the autophagosome with a bilayer membrane. The outer membrane of the autophagosome fuses with the lysosome to form the autolysosome and the cargo of the autophagosome is degraded by enzymes in the lysosome (Figure 1) [10].

Overall, the molecular mechanism of the autophagy process is carried out by autophagy-related genes (Atgs) and several critical complexes (Figure 1) [11]. Autophagy steps are hierarchically controlled by two complexes: namely the unc-51-like kinase 1 (ULK1) complex [10] and class III phosphatidylinositol 3-kinase (PtdIns(3)K) complex [12], as well as by two ubiquitin-like conjugation systems, namely the Atg8-lipid phosphatidylethanolamine (PE) and Atg12-Atg5 systems [13]. Firstly, the ULK1 serine threonine kinase complex (involving ULK1, FIP200, Atg13, and Atg101) regulates the onset of PAS by transducing the signals regulating the phosphorylation or recruitment of autophagosome formation proteins to induce autophagy [2,14]. Secondly, PAS is modified by phosphatidylinositol 3-phosphate (PtdIns(3)P) produced under the promotion of the phosphatidylinositol 3-kinase (PtdIns(3)K) complex containing the vacuolar protein sorting 34 (VPS34)/phosphatidylinositol 3-kinase catalytic subunit type 3 (PIK3C3), Atg6/Beclin1, Atg14/Barkor, VPS15/phosphoinositide-3-kinase regulatory subunit 4 (PIK3R4), and the fifth subunit nuclear receptor binding factor 2 (NRBF2)/Atg38 [15]. VPS34 plays an important role in the extension of autophagic vesicle membranes and the recruitment of Atg proteins to autophagic vesicles. Thirdly, two ubiquitin-like systems (a class of low molecular weight proteins to molecule undergo specific modifications to target proteins under the action of a series of specific enzymes), consisting of Atg5, Atg12, Atg16L, Atg7, Atg3, and Atg4, regulate the expansion of phagophore by lipidating the microtubule-associated protein light chain 3-I (LC3-I) to LC3-II [16]. The C-terminal glycine, when exposed to LC3 synthesized early and connected by Atg4, forms a cytoplasmic soluble LC3-I. After autophagy induction, LC3-I is conjugated with substrate PE on the surface of the autophagy membrane under the joint action of E1-like enzyme Atg7, E2-like enzyme Atg3, and the E3-like enzyme Atg5-Atg12-Atg16L complex to form membrane-binding LC3-II. As such, LC3-II is an important marker of autophagosomes, which increases with the increase in the autophagosome membrane. Atg9 may function by generating the highly curved vesicular intermediate, which is indispensable during phagophore growth [17]. WD-repeat protein interacting with phosphoinositide (WIPI), a kind of PI(3)P-binding autophagy protein [18], and FYVE (the PI(3)P sensor) [19] can directly bind to the phagophore membrane surface through its transmembrane domain to relegate the sensor to a ring around nascent phagophores. Fourthly, the endosomal sorting complexes required for transport (ESCRT) proteins, which function to constrict and sever narrow membrane necks, are recruited to close phagophores through interacting with PtdIns(3)P [20]. The next step is the fusion of the autophagosome with lysosome to break down the cargo. The soluble N-ethylmaleimide-sensitive factor attachment protein receptor (SNARE) proteins further promotes specific membrane fusion, forming Qa-, Qb-, Qc-, and R-SNARE. The Qa-SNARE syntaxin 17 (STX17) can be inserted into completed autophagosomes via its unusual C-terminal hairpin-like structure and mediates autophagosome–lysosome fusion by binding to its partner soluble NSF attachment protein 29 (SNAP29; Qbc-SNARE) and vesicle-associated membrane protein 8 (VAMP8; R-SNARE) [21]. Recently, it has been reported that ATG14 interacts with SNAP29 as well as the t-SNARE complex consisting of STX17 and SNAP29 in vitro, co-localizing with STX17 on the mature autophagosome directly [22]. After the autophagosome fuses with the lysosome, the last step is the degradation of the contents in the autolysosome. The autolysosome degrades macromolecules to nutrients for the cell and the targeted hydrolase is activated within the lysosome’s high acidic pH environment (between 4.5 and 5.0). Acidification of the lysosome is maintained by the lysosomal membrane proteins, such as lysosomal-associated membrane protein 1 (LAMP1) and LAMP2, and more importantly the vacuolar-type H (+)-ATPases (V-ATPases) [23]. Orchestration of Spns1 and the V-ATPase optimize acidification during autolysosomal biogenesis [21].

## 3. Sensing Mechanisms of Amino Acid Regulation Autophagy

Amino acids, as the building blocks of proteins, play a fundamental role in the environmental nutrient levels required for life [7]. When amino acids are scarce, proteins are degraded to replenish amino acid pools for responses to fluctuations via activating ubiquitination–proteasome degradation and autophagy in eukaryotes; hence, the precise sensing amino of acid levels may be used for the mobilization of autophagy. Regardless of the situation in which amino acid sensing occurs, the cell must be within the normal physiological range. Two evolutionarily conserved nutritional pathways occupy pivotal positions in amino acid sensing, namely mTORC1 and general control nonderepressible 2 (GCN2) pathways [24]. The mTORC1 pathway activation, via signal transduction, is unequally sensitive to different AAs. Regardless of amino acid specificity, GCN2 pathway inhibits transfer RNAs (tRNAs) from binding with AAs as a response to low levels of AA.

### 3.1. mTORC1 Pathway and Autophagy

**Figure 2:**
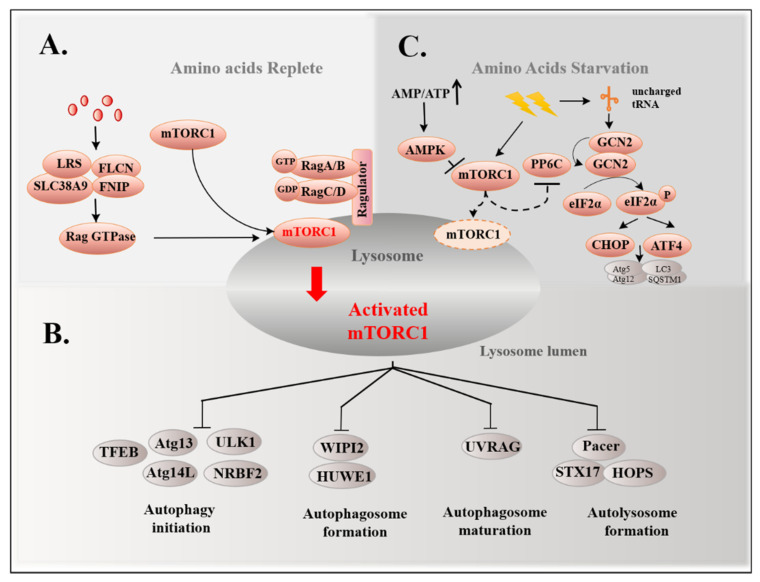
Classical amino acid sensing pathways regulate autophagy. (**A**) The abundant amino acids activate the Ragulator complex to promote the most active amino acid sensors, including SLC38A9, LRS, and FLCN-FNIP to RagGTPases, in which RagA/B binds to GTP and RagC/D to GDP, which recruits mTORC1 to the lysosome and is activated, thereby inhibiting autophagy. (The red arrow refers to the caption below, meaning the mTORC1 is activated in the lysosome.) (**B**) The mTORC1 activated by the abundant amino acids inhibits autophagy-related proteins, which are related with autophagy initiation, autophagosome formation, autophagosome maturation and autolysosome formation. (**C**) Amino acid starvation prevents the translocation of mTORC1 to the surface of the lysosome and inhibits its activity. GCN2 kinase binds to the elevated uncharged tRNA to be activated, then phosphorylates eIF2α and upregulates the transcriptional activities of ATF4 and CHOP to induce autophagy.

mTORC1 senses AAs via signal transduction. In the absence of amino acids or growth factors, mTORC1 is dispersed in the cytosol with a devitalized form [25]; however, under amino-acid-rich conditions, regulators such as the guanine nucleotide exchange factor (GEF) promote the process whereby the least active heterodimer of Rag A/B-GDP and Rag C/D-GTP convert to the most active form, in which Rag A/B bind to GTP and Rag C/D bind to GDP. The formation of Rag A/B-GTP and Rag C/D-GDP is capable of recruiting mTORC1 to the surface of the lysosome to be activated (Figure 2A) [25]. Rag GTPase is also a transfer station for amino acid sensors to mTORC1. Multiple amino acid sensors act on Rag GTPase to mediate the response of mTORC1 to amino acids, controlling the metabolic growth process, including solute carrier family 38, member 9 (SLC38A9), whose cytosolic N-terminal domain has a high affinity for the Rag-Ragulator [25] and leucyl-tRNA synthetase (LRS), switching on Rag D as a leucine sensor [26], as well as folliculin and its interacting partner (FLCN-FNIP), the GTPase-activating protein (GAP) of Rag C/D (Figure 2A) [27].

Here, mTORC1 regulates autophagy at different stages. Under nutrient-rich conditions, mTORC1 inhibits the synthesis of PI3P through phosphorylating several proteins and suppresses initial autophagy–membrane nucleation. These phosphorylated proteins include: ULK1, which is phosphorylated by mTORC1 at S757 [14]; Atg13-phosphorylation leading to disintegration of the ULK1 complex [28]; Atg14L, which is necessary for the assembly of the PtdIns(3)K complex [29]; NRBF2, a molecular switch of PtdIns(3)K [30]. Interestingly, instead of mTORC1 inhibition, amino acid starvation induces the dissociation of protein phosphatase 2A (PP2A) from inhibitory factor Alpha4, resulting in an increase in its phosphatase activity, which promotes dephosphorylation of the substrate ULK1 and induces autophagy [31]. Furthermore, mTORC1 phosphorylates WIPI2 at Ser395, enhancing the interaction with HUWE1 (HECT, UBA, WWE domain containing 1), an E3 ubiquitin ligase used for ubiquitination degradation of WIPI2, which inhibits autophagosome formation when rich in amino acids [32]. As far as we know, the biosynthesis of autophagosomes requires Atg5, Atg7, and LC3, while their inactivation is caused by mTORC1-dependent phosphorylation of P300, a histone acetyltransferase, at 4 serine residues in the C-terminal domain [33]. To inhibit the maturation of the autophagosome, UV radiation resistance-associated gene (UVRAG) can also be phosphorylated by mTORC1 [34]. Moreover, mTORC1 can also suppress autolysosome formation. In nutrient-rich states, rubicons such as autophagy enhancer (PACER) are directly phosphorylated by mTORC1 at Ser157, which disrupts the connection of PACER with the homotypic fusion and protein sorting (STX17-HOPS) complex. The STX17-HOPS complex is essential for autophagosome–lysosome tethering and fusion in late stages of autophagy [33]. These reports indicate that mTORC1 is involved in multi-stage autophagy regulation (Figure 2B). In fact, in addition to phase regulation of autophagy, mTORC1 is also able to control global autophagy flux by phosphorylating transcription factor EB (TFEB), a mediator of autophagy [35]. After a few years of research on the molecular characterization of mTORC1, mTORC1 regulates autophagy at different stages; however, mTORC1 is not equally sensitive to all amino acids produced by the Rheb guanosine triphosphatases (GTPase) for diverse signal transduction [36,37].

### 3.2. GCN2 Pathway and Autophagy

GCN2, an amino-acid-nonspecific serine–threonine protein kinase with high affinity to all uncharged tRNAs, senses intracellular amino acid levels through binding to uncharged tRNAs [38]; therefore, GCN2 can detect the overall amino acid level without the specific amino acid. In the protein synthesis machinery, tRNA combines with the ribosomes, forming a new peptide. The sensing process occurs during low amino acid levels, whereby the uncharged tRNA will accumulate at a low amino acid concentration and GCN2 binds to uncharged tRNAs through a domain homologous to histidyl tRNA synthetase, leading to homodimerization and autophosphorylation of GCN2 and subsequent activation [38]. Activated GCN2 phosphorylates the eukaryotic translation initiator factor 2α (eIF2α) at Ser51, which affects autophagy at the transcriptional level. The phosphorylation of eIF2α is able to upregulate activating transcription factor 4 (ATF4) and C/EBP-homologous protein (CHOP) to induce autophagy by enhancing the transcription of autophagy-related proteins. These proteins include Atg5, Atg12, LC3, and SQSTM1/p62 (Sequestosome 1), which are involved in initiation, elongation, and maturation of autophagosome [39]. Mouse models have demonstrated that knocking out GCN2 and ATF4 prevents the transcriptional activation of p62, an autophagic adapter that acts as a cargo receptor for degradation of ubiquitinated substrates, leading to leucine shortages in mammalian species [39,40]. Inhibition of GCN2 reduces LC3 accumulation when arginine is downregulated due to IFN-γ treatment in bovine mammary epithelial cells (BMECs) [41]. In HCT116 cells, GCN2 functions downstream of protopanaxadiol (PPD) to promote autophagy by upregulating the expression of Sestrin2 [42]. Interestingly, Sestrin2 interferes with translocation of mTORC1 to lysosomes [43], thereby inhibiting mTORC1 activity. In addition, GCN2 is activated by pharmacological inhibition of mTORC1, which requires the catalytic subunit of protein phosphatase 6 (PP6C) [44], suggesting that GCN2 pathways are related to enable autophagy to work (Figure 2C). Combined with the above, the regulation of mTORC1 is expected to act indirectly in the regulation of autophagy.

## 4. The mTORC1-Mediated Signal Transduction from Replete Functional Amino Acids to Autophagy

Amino acids, by activating the mammalian target of rapamycin (mTOR) kinase, regulate autophagy. It has been shown that autophagy is induced by GCN2 and mTORC1 pathways; as we all know how mTORC1 regulates autophagy, signal transduction of the mTORC1 pathway could be used for targets that are not equally sensitive to all amino acids. Amino acids are classically divided into essential and nonessential amino acids, according to whether they can be synthesized by the body itself. In addition, researchers define functional amino acids as those that participate in and regulate key metabolic pathways to improve the health of an organism and are also involved in the prevention and treatment of metabolic diseases, such as obesity, diabetes, and cardiovascular disease [45]; thus, from the mechanisms of major functional amino acids (e.g., arginine, leucine, glutamine and methionine) regulating mTORC1 through different signal transduction pathways, signal transduction pathways of FAAs are expected to be potential therapeutic targets for autophagy-related diseases in the future.

The discovery of the coordination of two sets of small GTPases, Rag and Ras homolog enriched in brain (Rheb), represents a breakthrough awareness of the role of mTORC1 in the response to amino acids and growth factors. The GTPase-activating proteins toward Rags 1 (GATOR1), which are anchored to the surface of the lysosome through the scaffold KICSTOR complex composed of KPTN, C12orf66, ITFG2, and SZT2-containing regulator of mTORC1 [46], act as a GAP of RagGTPases. Genetic epistasis analyses have shown that GTPase-activating proteins toward Rags 2 (GATOR2) may function as an upstream inhibitory factor for GATOR1, which indirectly activates mTORC1. To maximize the activation of mTORC1, the growth factor releases Rheb’s GAP dimerization and tuberous sclerosis (TSC)1/2 complex, promoting accumulation of Rheb-GTP on the surface of lysosomes, which changes mTORC1 to lysosomes and activates mTORC1 [47].

### 4.1. Multiple Arginine Sensors Signal Arginine to mTORC1

Arginine is the largest nitrogen-supplying amino acid. It is involved in protein synthesis and is the precursor of proline, glutamic acid and glutamic amine, and polyamine synthesis [48]. Animal dietary addition of arginine increased mTOR signaling activity, but how arginine regulates mTORC1 remained unclear until it was discovered that cellular arginine sensor for mammalian target of rapamycin complex 1 (CASTOR1) senses cytosolic arginine at a physiological concentration and binds to arginine [49,50]. CASTOR1 forms a homodimer or a heterodimer with CASTOR2, both of which can suppress GATOR2, causing downregulation of mTORC1. Arginine may directly bind to the two CASTOR1 complexes, releasing GATOR2, which removes the inhibition caused by GATOR2 and activates mTORC1 [51]. Structural analysis revealed that arginine binds to the two ACT (aspartate kinases, chorismate mutase, and TyrA) domains of CASTOR1, causing an allosteric regulation in the region adjacent to the GATOR2-binding site, which causes the dissociation of GATOR2 from CASTOR1 [49]. The generation of arginine in the lysosomal lumen by protein degradation processes such as autophagy is an important source of cytosolic arginine.

Besides CASTOR proteins, the uncharacterized human member 9 of the solute carrier family 38 (SLC38A9) is a putative sensor for arginine in the lysosomal lumen. SLC38A9 is located on the lysosomal membrane with two distinct domains: an 11-transmembrane segment for amino acid transportation and a cytosolic N-terminal domain consisting of 110 amino acids that is pivotal for interacting with the Rag GTPase-Ragulator complex [52]. Serving as an arginine-regulated amino acid transporter, SLC38A9 can transport some essential amino acids from lysosomes into the cytosol [53], and it is also necessary to transport leucine generated by lysosomal autophagy out of lysosomes to activate mTORC1 [25]. Loss of SLC38A9 inhibits activation of mTORC1 by arginine, indicating that SLC38A9 functions as a signal hub, sensing arginine availability for mTORC1 [54]; however, because of its unknown arginine-binding capacity and low affinity with arginine, SLC38A9 is not yet a bona fide arginine sensor [54].

**Figure 3:**
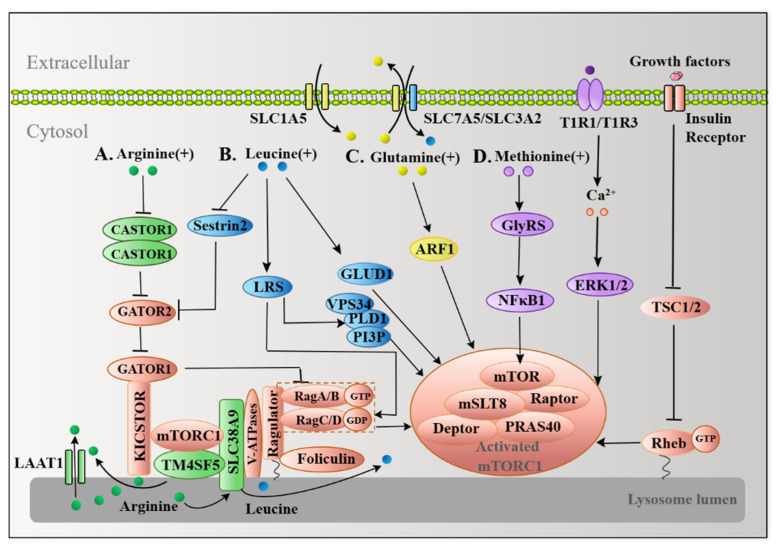
The mTORC1 mediates signal transduction of functional amino acids regulating autophagy; mTORC1 is an intracellular signal transfer station for amino acid regulation of mTORC1-dependent autophagy. (**A**) CASTOR1 senses and binds to arginine, which promotes the translocation and activation of mTORC1 to the lysosomes. SLC38A9 senses arginine in lysosomes and is required for the transfer of leucine out of lysosomes to activate mTORC1. The TM4SF5-mTORC1-SLC38A9 complex, together with LAAT1, facilitates the transfer of arginine out of lysosome. (**B**) Sestrin2 senses leucine in a manner similar to CASTOR1. LRS activates mTORC1 by activating RagGTPase or PLD1. The leucine metabolite acetyl-CoA acetylates the Raptor at K1097 via EP300. GLUD1 also contributes to the regulation of autophagy by leucine. (**C**) Arf1 GTPase participates in glutamine-mediated mTORC1 activation. (**D**) Methionine promotes the activation of GlyRS and activates NFκB1 to elevate the expression of mTORC1. T1R1/T1R3 senses extracellular methionine to stimulate ERK1/2 activation by enhancing the Ca^2+^ concentration and then activates mTORC1.

Transmembrane 4 L6 family member 5 (TM4SF5) serves as a membrane-based sensor that directly binds lysosomal arginine via its long extracellular loop (LEL). At physiological arginine concentrations, TM4SF5 moves to the surface of the lysosome, forming a complex with mTORC1, while SLC38A9 causes efflux of arginine [55]. CASTOR1, SLC38A9, and TM4SF5 specifically mediate arginine-activated mTORC1 and supply the arginine signaling network in autophagy control (Figure 3A).

### 4.2. Leucine Transmits Signals to mTORC1 in a Similar Manner to Arginine

Similar to arginine, leucine activates mTORC1 via several sensors. Sestrin2 is a recently reported leucine sensor in the mTORC1 pathway [56]. When leucine is insufficient, GATOR2 is suppressed by binding to Sestrin2. Inhibition of GATOR2 enhances the activity of GATOR1, which permits Rag A/B to maintain a GDP-binding form that inhibits mTORC1 and stimulates autophagy; however, when leucine levels increase, leucine binds to Sestrin2, dissociating Sestrin2 from GATOR2 and leading to upregulated activity of GATOR2 and inhibition of GATOR1, followed by activation of Rag A/B, which activates mTORC1 and inhibits autophagy at last [57]. HEK-293T cells expressing Sestrin2 mutants that are unable to bind leucine significantly reduce the localization of mTORC1 to the surface of lysosomes in the presence of leucine, indicating that when leucine activates mTORC1, binding of leucine to Sestrin2 is required [58]. Structural analysis also revealed that leucine binds to Sestrin2 through a lid–latch mechanism. The helices C2, C3, and C7 in the C-terminal domain of Sestrin2 form a single pocket that binds to leucine, and the charged residues Glu451 and Arg390 on both sides of the pocket anchor leucine through a salt bridge. The “lid” formed by a loop connecting helices C2 and C3 covers the top of leucine and His86 at the N-terminus forms a tight hydrogen bond with the C-terminal lid residue Thr375, which locks the lid on the bound leucine as a “latch”. Mutagenesis studies have shown that the N-terminal S190 and C-terminals Asp406 and Asp407 of Sestrin2 are all necessary for Sestrin2 combined with GATOR2 [59]. Importantly, Sestrin2 binds to GATOR2 at distinct sites from CASTOR1, which allows leucine and arginine to independently regulate mTORC1 [60].

LRS, a kind of aminoacyl-tRNA synthase, serves as another leucine sensor in the mTORC1 pathway. No enhancement of S6K phosphorylation by leucine was observed in the F50A/Y52A mutant of LRS, but it was clearly observed in the wide type of LRS, indicating that LRS is essential for leucine activation of mTORC1. Experiments involving co-immunoprecipitation and co-transfection demonstrated that LRS forms a complex with Raptor and Rag D in HEK 293T cells. The complex binds leucine and functions as the GAP for Rag D, which mediates leucine-induced activation of mTORC1 [26]. In addition, LRS mediates the activation of the amino-acid-induced Vps34-PLD1-mTORC1 pathway. Amino acids stimulate the production of PI3P by VPS34, through which PI3P interacts with the PX domain of phospholipase D1 (PLD1). The interaction with PI3P facilitates PLD1 translocation to the lysosome and promotes mTORC1 activation [61]. The Rag GTPase cycle is critical for mTORC1 to regulate autophagy. LRS acts as an “on” switch by hydrolyzing the GTP of Rag D, while Sestrin2 serves as an “off” switch through GATOR2 and GATOR1 to hydrolyze the GTP of Rag B [62].

Glutamate dehydrogenase (GLUD) can catalyze the dehydrogenation of glutamate and inhibit autophagy by suppressing the production of reactive oxygen species (ROS) and activating mTORC1. Knockdown of GLUD1 blocks the accumulation of GFP-LC3 puncta caused by leucine starvation, showing that GLUD1 is also essential for leucine-starvation-induced autophagy [63]. Sestrin2, LRS, and GLUD1 activate mTORC1 by leucine signaling transduction in autophagy control (Figure 3B).

### 4.3. Glutamine Activates mTORC1 in a RagA/B-Independent Fashion

As the most abundant free nonessential amino acid in the blood, glutamine accounts for about 20% of the total cytosolic amino acid pool. It has been reported that glutamine activates mTORC1 in a Rag A/B-independent fashion. In Rag A/B knockout mouse embryonic fibroblasts (MEFs) and HEK293A cells, glutamine is able to activate mTORC1 to a similar extent as control cells, uncovering the independence of Rag A/B in the regulation of glutamine on mTORC1. Glutamine promotes the localization of mTORC1 to lysosomes when knockout Rag A/B is present in MEF cells, which requires V-ATPase instead of Ragulator. Glutamine failed to activate mTORC1 in HEK293A Rag A/B KO cells that depleted adenosine diphosphate ribosylation factor-1 (Arf1) by interfering with RNA, indicating that Arf1 is required for glutamine to activate mTORC1 in Rag A/B knockout cells [64] (Figure 3C). Interestingly, bidirectional transport of glutamine and leucine is the key to activate mTORC1 and inhibit autophagy. This two-way system includes SLC1A5, which regulates L-glutamine uptake, and a bidirectional transporter, SLC7A5/SLC3A2, which simultaneously effluxes L-glutamine out of cells and transports essential amino acids (EAAs), including L-leucine, into cells. Depletion of glutamine or downregulation of SLC1A5 activates autophagy, indicating that both SLC1A5 and glutamine are negative regulators of autophagy [12].

### 4.4. Methionine Also Activates mTORC1 as a Sulfur-Containing Functional Amino Acid

Methionine is a sulfur-containing amino acid with a variety of biological functions, including protein metabolism, methylation, and oxidative stress. T1R1/T1R3 was confirmed as a methionine sensor in myotubes. T1R1/T1R3 is a G-protein-coupled receptor that directly senses the availability of extracellular amino acids and modulates mTORC1 activity through Ca^2+^ stimulation and activation of extracellular-signal-regulated kinases 1 and 2 (ERK1/2). Knocking down the T1R1/T1R3 receptor reduces the ability of amino acids to signal to mTORC1, which is embodied in the interference in mTORC1 localization so as to induce autophagy [65]. After the C2C12 cells are deprived of total amino acids, only a single re-addition of methionine can phosphorylate ribosomal protein S6 kinase 1 (S6K1), and mTORC1. Furthermore, T1R1/T1R3, as a sensor for methionine, is reported to participate in the regulation of mTORC1, since knockdown of T1R1 significantly decreases phosphorylation of ERK1/2, the Ca^2+^ levels, and activation of S6K1 induced by methionine in C2C12 cells [66].

Methionine promotes phosphorylation and nuclear translocation of Glycyl-tRNA synthetase (GlyRS) at Thr544 and Ser704. GlyRS is able to activate nuclear factor kappa B1 (NFκB1), followed by elevation of downstream mTORC1 expression levels (Figure 3D). In BMECs, after GlyRS knockout, the addition of methionine at a theoretical level of inhibition (0.6 mM) is not sufficient to inhibit autophagy, indicating that GlyRS is required for methionine inhibition of autophagy [67]. T1R1/T1R3 and GlyRS specifically mediate methionine to activate mTORC1 via the signaling network.

## 5. FAAs Starvation Induce Autophagy

FAAs activate mTORC1 through signal transduction, with it having been proven that mTORC1 is a key pathway to inhibit autophagy. Based on the existing cell types and animal evidence, it has been summarized that arginine, leucine, glutamine, methionine, and their metabolites regulate autophagy through other transductions, suggesting that FAAs could be potential clinical medicine pathways for autophagy-related diseases.

### 5.1. Arginine and Autophagy

**Figure 4:**
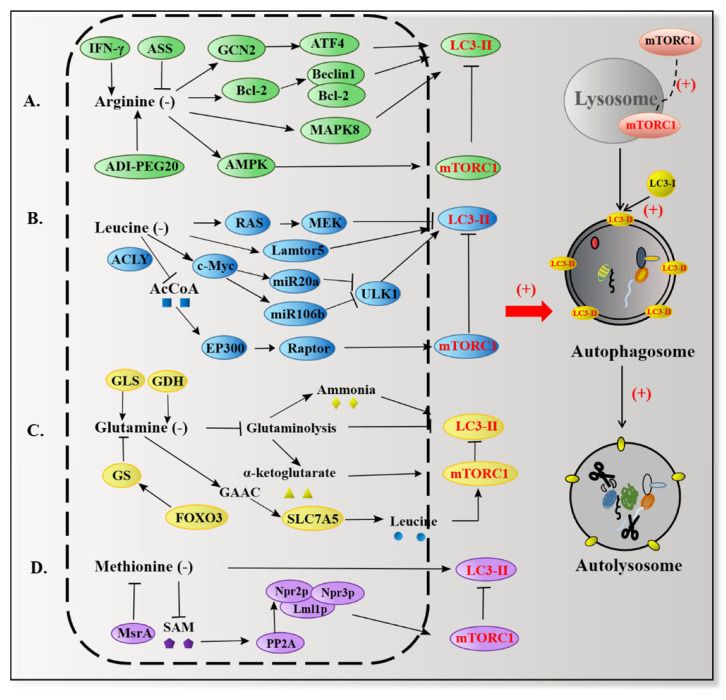
Specific amino acids regulate autophagy by inhibiting mTORC1 and increasing LC3-II expression. (The red arrow connects the left to right side of the diagram, representing the transition from mechanism to function; plus signs are used to present that amino acids promote autophagy.) (**A**) Arginine deficiency has been shown to upregulate autophagy via diverse pathways. (**B**) Leucine deficiency fails to induce autophagy in melanoma due to an overactivated RAS-MEK pathway. Leucine deficiency and its metabolite acetyl-CoA induce autophagy through mTORC1 in other cell types such as 293T cells and MEFs. (**C**) Glutamine-metabolizing enzymes GLS, GDH, and GS, which are enhanced by FOXO3 in Ba/F3 cells, are involved in glutamine synthesis and glutaminolysis. Glutaminolysis produces ammonia and α-ketoglutarate, ammonia promotes mTORC1-independent autophagy, and α-ketoglutarate inhibits mTORC1-dependent autophagy. Glutamine starvation upregulates SLC7A5 expression, increases intracellular uptake of leucine, activates mTORC1, and inhibits autophagy. (**D**) Knocking out MsrA increased p62-positive inclusion bodies and upregulated LC3-II expression in VSMC. SAM, synthesized from methionine, methylates PP2A to dephosphorylate the substrate Npr2p, thereby releasing the inhibition of mTORC1 by Npr2p, which suppresses NNS-autophagy.

Based on our increasingly clear understanding of arginine metabolism, arginine has been proven to occupy an important position in autophagy regulation (Figure 4A). Depletion of arginine can induce autophagy in a variety of cell types. In BMECs treated with 10 ng/mL interferon-gamma (IFN-γ) for 6, 24, and 48 h, the results showed that LC3 expression increased gradually with time and that inhibition of GCN2 expression significantly reduced the formation of LC3-II in the IFN-γ response. Meanwhile, IFN-γ promoted GCN2 phosphorylation in a time- and dose-dependent manner and arginine supplementation reversed GCN2 phosphorylation. This indicates that deprivation of arginine could induce autophagy in BMECs [41]. In MEF cells, depletion of arginine upregulates autophagy via GCN2-ATF4 signaling [68]. Toxoplasma infection depletes arginine in the host cell, resulting in phosphorylation of eIF2α by protein kinase GCN2 [69]. This will induce the transcriptional activity of ATF4 to promote autophagy through binding to a cyclic AMP response element binding site in the LC3-II promoter to upregulate LC3-II expression by using small interfering RNA (siRNA) and microarray analysis in cancer cells [70]. As a compensation mechanism, the upregulation of autophagy by ATF4 facilitates the uptake of arginine by enhancing the expression of the cationic amino acid transporter, CAT1 (SLC7A1) [63]. In human T lymphocytes, after transfection of EGFP-LC3 and incubation with arginine for 48 h, microscopy analysis showed that deficiency of arginine leads to endoplasmic reticulum stress and phosphorylates eIF2α, mitogen-activated protein kinases 8 (MAPK8), and Bcl-2, inducing the binding of Beclin1/Atg6 to Bcl-2, which promotes the formation of autophagosomes. Silencing inositol-requiring enzyme1α (IRE1α) expression can downregulate autophagy induced by arginine deprivation [71]. Autophagy induced by arginine deficiency may be a self-protection mechanism of cells in the early stages of stress. Most melanoma cells do not express aminosuccinate synthase (ASS), which blocks the synthesis of arginine from citrulline. The demand for arginine can only be met by exogenous supply in these cells. ADI-PEG20, an arginine deiminase, can degrade arginine. Studies have shown that arginine deficiency or ADI treatment can activate AMPK, inhibit mTORC1, and induce autophagy in ASS (−) melanoma cells. Silencing of Beclin1 will enhance cell death, suggesting that autophagy represents a survival strategy in ASS (−) melanoma cells [72]; however, the protective effect of autophagy may be an initial adaptation mechanism to arginine deprivation, because prolonging the time of arginine deprivation will impair the function of mitochondrial respiration and induce cytotoxic autophagy, at least partly leading to the death of ASS1-deficient breast cancer cells [73].

Arginine and its metabolites may have some roles in autophagy. L-arginine is well known to be the precursor of several metabolites, including nitric oxide (NO), polyamines, and ornithine [48]. NO induces apoptosis and inhibits autophagy in human hepatoma cells by disrupting the Beclin 1/VPS34 association and increasing the Bcl-2/Beclin 1 interaction, which may provide a novel strategy for the treatment of hepatocellular carcinoma (HCC) [74]. Polyamines, such as spermidine, limit brain damage and restore effective autophagy by dismantling misfolded proteins and dysfunctional mitochondria [75]; however, ornithine enhances AMPK mediated autophagy [76].

### 5.2. Leucine and Autophagy

In most cell types, leucine, rather than other branched chain amino acids, is the most potent amino acid that inhibits autophagy. Feeding mice with a diet supplemented with 3% casein caused an upregulation of autophagy, accompanied by accumulation of triglycerides (TG); however, the addition of leucine can inhibit autophagy by inhibiting the decrease in p62 and the increase in LC3 [77]. In HEK 293T cells, leucine deprivation increased the conversion of LC3-I to LC3-II along with a decline in p62, suggesting that leucine limitation induced autophagy. Furthermore, 10 mM leucine supplementation was able to restore autophagy activity to basal levels, while 3 mM leucine supplementation was sufficient to inhibit targeting of Barkor/ATG14 to autophagosomes. Moreover, rapamycin effectively inhibits the autophagy inhibitory effect of leucine addition and the addition of leucine does not inhibit autophagy activity in cells that deplete Rheb and Raptor. These findings indicate that leucine inhibition of autophagy is dependent on the mTORC1-Barkor pathway [78].

In addition to inhibition of mTORC1, leucine deficiency can also induce autophagy via indirect activation of ULK1. Global miRNA expression profiling screening combined with functional experiments showed that two members of the miR-17 microRNA family, miR-20a and miR-106b, negatively regulate the expression of ULK1 by affecting the stability of the ULK1 gene. Leucine deficiency can upregulate the activities of miR-20a and miR-106b via suppression of c-Myc, increase the expression of ULK1, and then induce autophagy in C2C12 myoblasts [79]. Dual-color DsRed-LC3-GFP reporter analysis demonstrated that leucine deprivation fails to induce autophagy in melanoma. This failure may be due to the overactivation of the RAS-MEK pathway in melanoma cells, which abolishes the inhibition of mTORC1 in leucine deficiency [80]. This suggests that the effect of leucine restriction on autophagy may depend on the type of cell and the physiological state of the cell. In the case of leucine starvation, knocking out Atg8 in Saccharomyces cerevisiae causes a slow death, while deletion of Atg11 elicits rapid cell death, indicating that the autophagy protein has both cytoprotective and cytocidal effects under leucine starvation conditions (Figure 4B) [81].

### 5.3. Glutamine and Autophagy

Stress conditions, such as disease, poor nutrition, or intense exercise, increase the body’s demand for glutamine. Glutamine plays a role in human health, including muscle growth, strengthening the immune system and participating in the synthesis of glutathione (an important antioxidant), which increases the body’s antioxidant capacity [82]. The addition of glutamine alone is sufficient to restore the activity of mTORC1 and inhibit autophagy. A metabolomics analysis demonstrated that glutamine reactivated mTORC1 by conversion to glutamate, while exogenously added ammonia could not replace glutamine. This indicates that the release of free amino acids from autophagy and glutamine jointly control the activity of mTORC1 in amino acid starvation [83]. After pharmacological inhibition of autophagy by inhibitors such as SAR405, the recovery of mTORC1 by glutamine is abolished, indicating that autophagy is necessary for glutamine reactivation of mTORC1. Moreover, when glutamine is deficient, autophagy provides glutamine in sub-mm concentrations, maintains mitochondrial oxidative phosphorylation, and promotes cell survival [84]. Interestingly, glutamine starvation has also been shown to inhibit autophagy. Glutamine starvation activates the general amino acid control (GAAC) pathway, upregulates SLC7A5, increases intracellular uptake of leucine, activates mTORC1, and inhibits autophagy, which may be a means of preventing excessive autophagy in cells under starvation conditions [85].

The synthesis of glutamine is also closely related to autophagy. Three forkhead box (FOX) O transcription factors were negatively regulated by PI(3)K-PKB (also called Akt) in mammals, namely FOXO3, FOXO4, and FOXO1. Their activation is related to cell growth and stress resistance. Research has shown that activation of FOXO3 in Ba/F3 cells upregulates the expression of glutamate synthetase (GS), which was accompanied by an increase in glutamine levels. Upregulated glutamate synthetase further leads to inhibition of mTORC1 and increased autophagy [86], as measured by LC3 lipidation in a variety of cell lines, including MSCs, U2OS, and Ba/F3 cells. MSO, an irreversible inhibitor of glutamine synthetase activity [87], abolished the increase in the WIPI-1 puncta by FOXO3 activation, indicating that glutamate synthetase is not only important for autophagy at a physiological level, but also for autophagy induced by starvation. Collectively, glutamine synthetase can induce autophagy to promote cell survival [88].

Glutamine metabolism is carried out by a process named glutaminolysis, which involves two steps: firstly, glutaminase (GLS) catalyzes the conversion of glutamine to glutamate; secondly, glutamate dehydrogenase (GDH) promotes the conversion of glutamate to α-ketoglutarate. Silencing of GLS and GDH blocked the inhibition of autophagy by leucine and glutamine, revealing that glutaminolysis inhibits autophagy. This view was further confirmed by the increased levels of endogenous LC3-II after the addition of 6-diazo-5-oxo-L-norleucine (DON), a GLS inhibitor, to pharmacologically inhibited glutaminolysis [89]. The regulation of glutaminolysis on autophagy is mainly determined by the products after degradation. On the one hand, glutamine is converted to α-ketoglutarate, which stimulates binding of RAG to GTP and leads to activation of mTORC1, which inhibits autophagy [90]. On the other hand, glutamine catabolizes into ammonia, which promotes mTORC1-independent autophagy and in turn protects cells from tumor necrosis factor-α (TNF-α)-induced death of U2OS osteosarcoma cells [91] (Figure 4C).

### 5.4. Methionine and Autophagy

A lack of methionine reduces the phosphorylation of S6K1 and affects cell growth. In pig intestinal epithelial cells infected with enterotoxigenic *Escherichia coli* (ETEC), methionine deprivation exacerbates cytotoxicity and accelerates death, which may be due to defects in autophagy [92], suggesting that the level of methionine is closely related to autophagy. Methionine restriction effectively induces autophagy, increases acidification of the vacuole, and extends the life of the yeast cells [93]. As an antioxidant repair protein, methionine sulfoxide reductases (Msrs) are capable of converting oxidized methionine sulfoxide to methionine. The defective methionine sulfoxide repair is used as a functional research model for methionine oxidation. Oxidation of methionine, which is manifested by the absence of methionine sulfoxide reductase A (MsrA), causes upregulation of autophagy. Knocking out MsrA in mouse aortic smooth muscle cells increases p62-positive inclusion bodies and upregulates LC3-II expression [94].

Methionine can be converted to S-adenosylmethionine (SAM) via the action of SAM synthetase. SAM is the sole methyl donor for many methyltransferases. Protein methylation is an important post-transcriptional modification that is closely related to the formation of autophagosomes and the expression of autophagy-related proteins [95]. Transferring yeast from a nutrient-rich medium to a minimal nutrient medium using lactate as a carbon source still induces mitosis and general autophagy. Sutter et al. termed this autophagy no nitrogen starvation-induced autophagy (NNS-autophagy). In essence, this is a form of autophagy that deals with milder environmental changes [96]. Importantly, a complex of three proteins, Iml1p/Npr2p/Npr3p, is required for NNS autophagy. Lml1p binds to phosphorylated Npr2p to co-localize to PAS to promote NNS-autophagy. Npr2p/Npr3p inhibits mTORC1 to prevent cell overgrowth [97].

There is a single amino acid that specifically inhibits NNS autophagy—methionine. A series of metabolic analyses have found that methionine and its downstream metabolite, SAM, play a key role in inhibition of NNS autophagy. Methionine is metabolized to SAM, which provides methyl for Ppm1p, the only link between methionine and PP2A that regulates autophagy. Ppm1p transfers the methyl provided by SAM to PP2A and catalyzes the carboxy terminal leucine residue of the C subunit of PP2A. Methylation of PP2A dephosphorylates the substrate Npr2p, thereby inhibiting NNS-autophagy (Figure. 4D) [98]. This observation suggests that methionine and its metabolite SAM are sentinel metabolites that are significantly reduced in the face of reduced nutrient levels that control autophagy [98]. Among this, PP2A serves as a methionine sensor.

## 6. FAAs as Potential Treatments for Autophagy-Related Diseases

Functional amino acids control autophagy by regulating mTORC1 and other pathways [99], which can be used as treatment method for related autophagy diseases, such as cancer, human neurodegeneration, type II diabetes, cardiovascular disorders, mitochondrial disease, inflammatory, and obesity [100]. There are less studies on the mechanisms of functional amino acids in the treatment of autophagy-related diseases. Due to the increased studies on the regulation of autophagy by functional amino acids in recent years, it is expected that specific amino acids will be found that serve as therapeutic targets for autophagy-related diseases. (Table 1).

### 6.1. Cancer

The exact roles of autophagy are context-dependent in the progression of cancer. The cytoprotective specialty of autophagy is proven to have anticancer effects before tumorigenesis because defective autophagy increases the risk of cancer; however, during the growth of primary tumors, autophagy promotes the growth of tumor cells by degrading cargo to fuel cellular metabolism. Intriguingly, the induction of autophagy is a side effect of cancer treatment, while inhibition of autophagy seems to be a valid strategy to enhance the therapeutic efficacy. Conversely, studies have also reported that autophagy can participate in the death of immunogenic cells, thereby exerting anticancer function. Autophagy promotes the release of ATP [121] and high-mobility group box-1 (HMGB1) [122] from dying tumor cells, which recruit immune effectors to trigger tumor-specific immune responses. P53 may be involved in the modulation of autophagic bidirectional function of pancreatic ductal adenocarcinoma in mice. When P53 is present, loss of autophagy blocks intraepithelial neoplasias, whereas in the absence of P53, suppression of autophagy promotes tumor proliferation [123].

Functional amino acids are related to cancer development. Tumor growth is inseparable from arginine metabolism. A lack of aminosuccinate synthase1 (ASS1) is a vulnerability of cancer cells, known as arginine auxotrophy. Dietary arginine restriction inhibits ASS1-deficient breast cancer cells. In addition, arginine starvation kills tumor cells by inducing asparagine synthetase (ASNS), depleting aspartate in these cells to promote cytotoxicity [102]. Arginine deiminase (ADI) is reported to be a new approach for treating prostate cancer by inducing autophagy, which indicates that autophagy is involved in the regulation of cancer by arginine metabolism [124]. Methionine has been proven to be a nexus of cancer medicine and amino acid nutrition. In a mouse model of triple-negative breast cancer (TNBC), the efficacy of lexatumumab is enhanced through combination with methionine restriction (MR) [102]. Glioma is highly dependent on methionine and cystine. Studies have shown that the methionine and cystine double deprivation enhances the level of ROS to induce autophagy, thereby inhibiting the proliferation of glioma in vivo and in vitro [103]. Another functional amino acid, glutamine, is also considered to be an essential amino acid for KRas-driven cancer cells. Blocking the utilization of glutamine causes S phase stagnation in KRas-driven cancer cells [104]. These findings reveal that the regulation of autophagy by amino acids may serve as a new strategy to treat cancer.

### 6.2. Aging

Aging, an irreversible biological process, is a risk factor for many diseases, including cardiovascular disease and type 2 diabetic mellitus (T2DM). Extensive evidence suggests that the ability for autophagy gradually declines with age. Aging has been shown to be accompanied by a reduction in multiple ATGs of rodents and drosophila [125]. Furthermore, in normal aging humans, the expression levels of BECN1, Atg5, and Atg7 are decreased compared with young people [126]. This suggests that the failure of autophagy associated with aging is conserved across diverse organisms [127]. In view of this, autophagy activation is used as a new way to alleviate diseases of the aging process. Caloric restriction (CR), limiting nutrient intake without malnutrition, is an effective intervention to delay aging. Moreover, CR mimetics (CRM), mimicking the biological functions of CR, also through a variety of nutrient sensing pathways, including mTOR, AMPK, and Sirtuins, stimulates autophagy [128].

Aging can also promote cardiovascular diseases and activation of autophagy can effectively protect the heart in the aging process and relieve diseases such as atherosclerosis, coronary artery disease, and arrhythmia [129,130]. L-arginine affects the cardiovascular system by producing NO. Experiments in rabbits treated with high-fat diets clearly showed that L-arginine alleviates atherosclerosis by reducing NO synthesis [105]. Oral L-arginine supplements can lower blood pressure, although the exact mechanism of this effect is not fully understood [106].

Leucine slows aging-related diseases by promoting the synthesis of muscle proteins. mTORC1 is believed to be the convergence point of the leucine-mediated mRNA translation initiation effect, and is a molecular target for preventing muscle loss [131,132]. Leucine stimulates muscle protein synthesis in vitro and in vivo in mouse models, with many potential applications in health and disease as a medicinal nutrient used to stimulate the release of endogenous insulin, in addition to inhibiting the breakdown of muscle protein. For example, leucine administration affects type 2 diabetes; by observing patients with long-term type 2 diabetes, a combination of protein and leucine can increase the insulin response to carbohydrate intake by two to four times [107]. Recently, studies have shown that intake of leucine can strengthen the elderly intake of protein and amino acids in muscle protein synthesis passivation [133]. Supplementation of leucine is responsible for the anabolism of muscle protein synthesis and the current recommended intake is 55 mg kg^−1^ [134]. Part of the immunoglobulin superfamily, Wnt signaling promotes muscle regeneration, reducing the level of LC3-labeled Dvl2 and preventing cells from undergoing autophagy in Islr KO mice [135]. Leucine can strongly stimulate skeletal muscle anabolism and overcome the anabolism resistance of aging [134]. In addition, leucine can promote the protein synthesis of skeletal muscle and the transformation of muscle fiber types from fast muscle to slow muscle [136].

In addition, the exposure of human umbilical vein endothelial cells to pharmacological concentrations (0.5 mmol/L) of L-arginine for 7 days accelerates senescence by detecting elevated levels of senescence-associated beta-galactosidase (SA-β-gal) and the expression of vascular cell adhesion molecule-1 (VCAM-1) and intercellular adhesion molecule-1 (ICAM-1); however, silencing S6K1 or arginase-II abolishes the enhancement, while the treatment of rapamycin, an inhibitor of mTORC1, would also significantly reduce the level of SA-β-gal, suggesting that positive crosstalk between S6K1 and arginase-II mediates the aging effects of arginine [137]. The depletion of a single amino acid, methionine, prolongs the lifespan of model organisms from yeast to rodents, which has been shown to require autophagy involvement. Knocking out the necessary autophagy-related gene Atg5, Atg7, or Atg8 abolishes the longevity-enhancing capacity of MR in yeast. Furthermore, the disruption of the vacuolar ATPase interrupts the lifespan prolongation of MR, demonstrating that vacuolar acidification is also necessary [93]. Lipid and bile acid metabolism have also been found to be associated with the life extension effects of MR. LmnaG609G/G609G mutation mice exhibit pathological features of progeria, named Hutchinson–Gilford progeria syndrome (HGPS), accompanied by disorders of lipid metabolism, such as glycerophospholipid, linoleic and alpha-linolenic acid metabolism, and the loss of primary bile acids. Dietary methionine restriction with a low concentration (0.12%) of methionine is able to extend life and restore the lipid profile and level of bile acids in LmnaG609G/G609G mice. Although specific mechanisms remain to be elucidated, methionine restriction is an effective means of treating aging-related diseases in a variety of species [138].

### 6.3. Obesity

Obesity is characterized by the accumulation of dysfunctional adipose tissue, which triggers metabolic stress by releasing pro-inflammatory adipokines, such as leptin and TNF, as well as increasing levels of fatty acids, LDL cholesterol, and triglycerides. Obesity threatens human health and increases the prevalence of multiple complications, such as insulin resistance, diabetes mellitus, and inflammation. Current treatments for obesity focus primarily on energy intake restriction, although the effects are limited. Importantly, autophagy has been reported to affect obesity. This reveals a new strategy for treating obesity and its complications through altered autophagy. Liver-specific knockdown of the autophagy genes Atg7 and TFEB promotes hepatic steatosis and weight gain; however, the pathological phenotype will be rescued by the overexpression of Atg7 and TFEB [139]. Specific knockdown of Atg7 in adipose tissue reduces the weight of white adipose tissue (WAT) and improves insulin resistance (IR), revealing tissue specificity in autophagy-regulated obesogenesis [140]. Attenuated autophagic clearance of adipocytes has been shown to be associated with downregulation of death-associated protein kinase 2 (DAPK2) in obese patients [141]. This suggests that the recovery of autophagy function may be an effective strategy for the treatment of obesity.

Branched chain amino acids (BCAAs) promote lipolysis of adipose tissue, as inhibition of sterol regulatory element binding protein-1c (SREBP-1c) was observed, which caused liver damage in high-fat diet (HFD)-induced obese mice. Mechanism studies suggest that this may be due to the inhibition of hepatic autophagy by activated mTORC1 [142]. Studies have shown that the addition of dietary arginine can promote lipolysis. Clinical evidence also has shown that dietary arginine supplementation can reduce obesity, lower arterial blood pressure, increase antioxidants, normalize endothelial dysfunction, and mitigate type 2 diabetes [108]. In clinical trials of obese patients, the supplementation of 8.3 g/d L-arginine significantly reduced bodyweight and adipose tissue weight. In a genetic analysis, L-arginine upregulated the expression of nitric oxide synthase-1, AMPK, and heme oxygenase-3 in adipose tissue to facilitate lipolysis [109]. Arginine may promote the hydrolysis of porcine adipocytes by upregulating the expression levels of adipose triglyceride lipase (ATGL) and hormone-sensitive lipase (HSL). The quality of pig muscle depends on the lipid deposition pattern in the muscle, so the addition of arginine in the diet can be used to prevent excessive lipid deposition in pigs [110,111].

Hyperglycemia, a form of obesity complication, has also been shown to be affected by glutamine. Glutaminolysis promoted by activation of mitochondrial α-ketoglutarate dehydrogenase can promote the production of glucose. This is due to the contribution of glutamine to the carbons of glucose during gluconeogenesis. Mechanistically, the glutamine–glucose flux of mice with decreased levels of glutaminase 2 (GLS2), a glutamate-metabolizing enzyme, decreased, indicating that GLS2 promoted the contribution of glutaminolysis to glucose production [112]. In short, functional amino acids give a new understanding for the treatment of obesity.

### 6.4. Immune Metabolism Disorder

Immune metabolism plays an important role in health and disease, affecting the occurrence and development of obesity, type II diabetes, cardiovascular disease, and tumors. In recent years, it has been found that the immune metabolism disorder has a great influence on the function and differentiation of immune cells, causing inflammation. In the resting state, immune cells maintain a state of low energy consumption, although when the microenvironment of the immune cells changes, in order to adapt to environmental changes and play their roles, the metabolic level changes, which is related to autophagy. It has been demonstrated that T cell receptors are activated by antigens and exert effects through co-stimulation of mTOR-dependent processes. Activated T cells undergo multiple cell divisions and convert glucose and glutamine into biological substances, a process that requires the uptake of large amounts of amino acids and relies on TORC1 pathways [143]. The main regulation of immune metabolism occurs under the action of mTORC1, which activates lysosome and induces autophagy. For example, Tregs depend on the level of grading of mTORC1 activity. In the absence of mTORC1 function, Tregs fail to inhibit effector T cells, leading to severe autoimmune disease. Inhibitors of mTORC1 in systemic lupus erythematosus (SLE) show great promise and direct inhibition of glycolysis can also prevent the disease, while the inhibitors have not been found to have extensive cytotoxicity clinically [144]. T cell metabolic adaptation diseases can be combined with current immunotherapy approaches through mTOR suppression and the use of antidiabetic drugs such as metformin.

Deficiency of functional amino acids promotes remodeling and programming of metabolic tissues [145]. Moderate restriction of protein can improve metabolic healthy BCAA in mice and humans and effectively prolong the mean survival time of patients with liver cirrhosis. In the mouse model of liver cirrhosis, BCAA supplementation enhanced the activity of liver CD8^+^ T cells. This may have the potential to inhibit the occurrence of liver cancer [146]. C57BL/6J wild-type mice on a 7% calorie-derived protein diet showed improved glucose tolerance, while 19 male subjects showed similar results. Leucine restriction can reduce inflammation by inhibiting NLRP3 inflammasome-mediated inflammation and reducing Th17 differentiation via amino acid sensing of GCN2 [113]. Monocytes and macrophages increase SLC7A5 expression and leucine uptake under lip-polysaccharide (LPS) induction. SLC7A5-deficient T cells, being unable to take up leucine, activate mTORC1 and MyC-dependent glycolysis, as they are unable to respond to antigens for metabolic reprogramming [115]. Regulation of glutamine uptake is an indispensable step in energy production in the activation of T cells [115]. T cells cultured in the absence of glutamine cannot proliferate or produce IL-2 or IFN-γ [116]. Glutaminase 1 (GLS1) inhibitor inhibited Th17 cell differentiation and improved experimental autoimmune encephalomyelitis (EAE) in mice [117]. In autoimmune arthritis in SKG mice, Gls1 inhibition reduced the number of proliferative Ki-67-positive synovial cells and alleviated arthritis [118]. FAAs may be used as a new treatment for immune metabolism disorder.

### 6.5. Neurodegeneration

Neurodegenerative diseases, including Alzheimer’s disease, Parkinson’s disease, and Huntington’s disease, are chronic progressive cognitive impairment diseases. These diseases are caused by irreversible degradation of neurons, excessive proliferation of glial cells, and accumulation of some abnormal proteins in the nerve cells to produce cytotoxicity and cause dysregulation of cells. Neurodegenerative diseases are often accompanied by the downregulation of autophagy [40]. In this context, autophagy proteins serve as biomarkers of the disease. P62 binds to ubiquitin and plays a key role in cell clearance. P62-positive inclusion is also used as a diagnostic marker for several neurodegenerative diseases [40]. Studies of functional amino acids and the nervous system are currently focused on excitatory amino acids as neurotransmitter roles. For example, glutamate is an excitatory neurotransmitter, although it is also a neurotoxin. Excessive accumulation can lead to the death of nerve cells and is related to the above-mentioned pathogenesis of neurodegenerative diseases. Excitatory amino acid transporter (EAAT)-2 has been shown to control the concentration of glutamate in the synaptic cleft [120]. Knockout of GLUD1 in the central nervous system leads to defects in redox metabolism of astrocytes, although the level of glutamate in the brain does not change, indicating that glutamate is at the crossroad of glutamate metabolism and neurotransmitter pathways [66]. Glutamine synthetase 1 (GS1) increases autophagy degradation of mutated huntingtin aggregates in neurons and improves the motility of Huntington’s disease in a drosophile model [121].

## 7. Conclusions

Collectively, autophagy is a key way for cells to adapt to the challenges of nutritional stress and to maintain cell homeostasis. The process of autophagy is regulated closely by amino acids. Functional amino acids, such as arginine, leucine, glutamine, and methionine regulate autophagy through their respective signal transduction mechanisms. Furthermore, mTORC1 serves as the main signal transfer station. Functional amino acids affect the development of many autophagy-related diseases, including cancer, neurodegenerative diseases, aging, obesity, and their complications. More research is needed to explore the link between amino acid metabolism and autophagy and to elucidate the mechanisms of metabolic physiological processes and disease responses.

## Figures and Tables

**Table 1:** Specific amino acids may serve as therapeutic targets for autophagy-related diseases.

Disease Types	Amino Acids Types	Key Findings	Reference
Cancer	Arginine	Restriction inhibits ASS1-deficient breast cancer cells	[101]
	Methionine	Restriction inhibits breast cancer and glioma	[102,103]
	Glutamine	Restriction causes S phase stagnation in KRas-driven cancer cells	[104]
Cardiovascular	Arginine	Supplements alleviate atherosclerosis and lowers blood pressure	[105,106]
Type 2 diabetes	Leucine	Increases the insulin response	[107]
Obesity	Arginine	Supplements and promotes lipolysis and reduces body/adipose tissue weight	[108,109,110,111]
	Glutamine	Supplements and promotes the production of glucose	[112]
Immune metabolism disorder	Leucine	Restriction reduces inflammation by inhibiting NLRP3t	[113,114]
	Glutamine	Restriction activates T cells and produces IL-2 or IFN-γ	[115,116,117,118]
Neurodegeneration	Glutamine	Glutamate is a neurotoxin	[119,120]

## Abbreviations

ATF4	activating transcription factor 4
ATGL	adipose triglyceride lipase
AS	aminosuccinate synthase
ASS1	aminosuccinate synthase1
ADI	arginine deiminase
As2O3	arsenic trioxide
ASNS	asparagine synthetase
ACT	aspartate kinases, chorismate mutase and TyrA
Atgs	autophagy-related genes
BMECs	bovine mammary epithelial cells
BCAAs	branched chain amino acids
CHOP	C/EBP-homologous protein
CR	caloric restriction
CASTOR1	cellular arginine sensor for mammalian target of rapamycin complex 1
CMA	chaperon-mediated autophagy
CRM	CR mimetics
Dvl2	dishevelled-2
ESCRT	endosomal sorting complexes required for transport
ETEC	Escherichia coli
EAAs	essential amino acids
eIF2α	eukaryotic translation initiator factor 2α
EAAT	excitatory amino acid transporter
EAE	experimental autoimmune encephalomyelitis
FOX	forkhead box
FAAs	functional amino acids
GAAC	general amino acid control
GCN2	general control nonderepressible 2
GLUD	glutamate dehydrogenase
GDH	glutamate dehydrogenase
GS	glutamate synthetase
GLS	glutaminase
GLS1	glutaminase 1
GLS2	glutaminase 2
GS1	glutamine synthetase 1
GEF	guanine nucleotide exchange factor
HCC	hepatocellular carcinoma
HFD	high-fat diet
HMGB1	high-mobility group box-1
Hcy	homocysteine
HOPS	homotypic fusion and protein sorting
HSL	hormone-sensitive lipase
HGPS	Hutchinson–Gilford progeria syndrome
H2S	hydrogen sulfide
Islr	immunoglobulin superfamily containing leucine-rich repeat
IRE1α	inositol-requiring enzyme1α
ICAM-1	intercellular adhesion molecule-1
IFN-γ	interferon-gamma
LRS	leucyl tRNA synthetase
LC3-I	light chain 3-I
LPS	lip-polysaccharide
LEL	long extracellular loop
LAMP1	lysosomal-associated membrane protein 1
mLST8	mammalian lethal with SEC13 protein 8
mTORC1	mammalian target of rapamycin complex 1
MET	methionine
MR	methionine restriction
MsrA	methionine sulfoxide reductase A
Msrs	methionine sulfoxide reductases
MAPK8	mitogen protein kinases 8
MEF	mouse embryonic fibroblast
MEFs	mouse embryonic fibroblasts
NO	nitric oxide
NNS-autophagy	non-nitrogen-starvation-induced autophagy
NFκB1	nuclear factor kappa B1
NRBF2	nuclear receptor binding factor 2
PE	phosphatidylethanolamine
PtdIns(3)K	phosphatidylinositol 3-kinase
PtdIns3P	phosphatidylinositol 3-phosphate
PLD1	phospholipase D1
PAS	pre-autophagosome structures
PRAS40	proline-rich Akt substrate of 40 kDa
PP2A	protein phosphatase 2A
PPD	protopanaxadiol
Raptor	ragulatory-associated protein of mTOR
ROS	reactive oxygen species
S6K1	ribosomal protein S6 kinase, polypeptide 1
PACER	rubicon like autophagy enhancer
SAM	S-adenosylmethionine
SA-β-gal	senescence-associated beta-galactosidase
SQSTM1/p62	sequestosome 1
siRNA	small interfering RNA
SNARE	soluble N-ethylmaleimide-sensitive factor attachment protein receptor
SNAP29	soluble NSF attachment protein 29
SLC38A9	solute carrier family 38, member 9
SREBP-1c	sterol regulatory element binding protein-1c
STX17	syntaxin 17
SLE	systemic lupus erythematosus
TFEB	transcription factor EB
TM4SF5	transmembrane 4 L Six Family Member 5
TNBC	triple-negative breast cancer
TNF-α	tumor necrosis factor-α
T2DM	type 2 diabetic mellitus
ULK1	unc-51-like kinase 1
UVRAG	UV radiation resistance-associated gene
VPS34	vacuolar protein sorting 34
VCAM-1	vascular cell adhesion molecule-1
VAMP8	vesicle-associated membrane protein 8
WIPI	WD-repeat protein interacting with phosphoinositides
WAT	white adipose tissue
HUWE1, HECT, UBA	WWE domain containing 1

## References

[1] Radewa J. (1963). Observations on autophagocytosis phenomena in the blood. Z. Rheumaforsch..

[2] Levine B., Kroemer G. (2019). Biological Functions of Autophagy Genes: A Disease Perspective. Cell.

[3] Galluzzi L., Bravo-San Pedro J.M., Levine B., Green D.R., Kroemer G. (2017). Pharmacological modulation of autophagy: Therapeutic potential and persisting obstacles. Nat. Rev. Drug Discov..

[4] Mortimore G.E., Schworer C.M. (1977). Induction of autophagy by amino-acid deprivation in perfused rat liver. Nature.

[5] He L., Zhang J., Zhao J., Ma N., Kim S.W., Qiao S., Ma X. (2018). Autophagy: The Last Defense against Cellular Nutritional Stress. Adv. Nutr..

[6] Nicklin P., Bergman P., Zhang B., Triantafellow E., Wang H., Nyfeler B., Yang H., Hild M., Kung C., Wilson C. (2009). Bidirectional transport of amino acids regulates mTOR and autophagy. Cell.

[7] He L., Eslamfam S., Ma X., Li D. (2016). Autophagy and the nutritional signaling pathway. Front Agr. Sci. Eng..

[8] Gwinn D.M., Shackelford D.B., Egan D.F., Mihaylova M.M., Mery A., Vasquez D.S., Turk B.E., Shaw R.J. (2008). AMPK phosphorylation of raptor mediates a metabolic checkpoint. Mol. Cell.

[9] Parzych K.R., Klionsky D.J. (2014). An overview of autophagy: Morphology, mechanism, and regulation. Antioxid. Redox Signal.

[10] Feng Y., He D., Yao Z., Klionsky D.J. (2014). The machinery of macroautophagy. Cell Res..

[11] Shpilka T., Weidberg H., Pietrokovski S., Elazar Z. (2011). Atg8: An autophagy-related ubiquitin-like protein family. Genome Biol..

[12] Russell R.C., Tian Y., Yuan H., Park H.W., Chang Y.Y., Kim J., Kim H., Neufeld T.P., Dillin A., Guan K.L. (2013). ULK1 induces autophagy by phosphorylating Beclin-1 and activating VPS34 lipid kinase. Nat. Cell Biol..

[13] Ohsumi Y. (2001). Molecular dissection of autophagy: Two ubiquitin-like systems. Nat. Rev. Mol. Cell Biol..

[14] Kim J., Kundu M., Viollet B., Guan K.L. (2011). AMPK and mTOR regulate autophagy through direct phosphorylation of Ulk1. Nat. Cell Biol..

[15] Cao Y., Wang Y., Abi Saab W.F., Yang F., Pessin J.E., Backer J.M. (2014). NRBF2 regulates macroautophagy as a component of Vps34 Complex I. Biochem. J..

[16] Geng J., Klionsky D.J. (2008). The Atg8 and Atg12 ubiquitin-like conjugation systems in macroautophagy. ‘Protein modifications: Beyond the usual suspects’ review series. EMBO Rep..

[17] Noda T., Kim J., Huang W.P., Baba M., Tokunaga C., Ohsumi Y., Klionsky D.J. (2000). Apg9p/Cvt7p is an integral membrane protein required for transport vesicle formation in the Cvt and autophagy pathways. J. Cell Biol..

[18] Proikas-Cezanne T., Takacs Z., Dönnes P., Kohlbacher O. (2015). WIPI proteins: Essential PtdIns3P effectors at the nascent autophagosome. J. Cell Sci..

[19] Jang D.J., Lee J.A. (2016). The roles of phosphoinositides in mammalian autophagy. Arch. Pharm. Res..

[20] McCullough J., Colf L.A., Sundquist W.I. (2013). Membrane fission reactions of the mammalian ESCRT pathway. Annu. Rev. Biochem..

[21] Itakura E., Kishi-Itakura C., Mizushima N. (2012). The hairpin-type tail-anchored SNARE syntaxin 17 targets to autophagosomes for fusion with endosomes/lysosomes. Cell.

[22] Diao J., Liu R., Rong Y., Zhao M., Zhang J., Lai Y., Zhou Q., Wilz L.M., Li J., Vivona S. (2015). ATG14 promotes membrane tethering and fusion of autophagosomes to endolysosomes. Nature.

[23] Mindell J.A. (2012). Lysosomal acidification mechanisms. Annu. Rev. Physiol..

[24] Darnell A.M., Subramaniam A.R., O’Shea E.K. (2018). Translational Control through Differential Ribosome Pausing during Amino Acid Limitation in Mammalian Cells. Mol. Cell.

[25] Wyant G.A., Abu-Remaileh M., Wolfson R.L., Chen W.W., Freinkman E., Danai L.V., Vander Heiden M.G., Sabatini D.M. (2017). mTORC1 Activator SLC38A9 Is Required to Efflux Essential Amino Acids from Lysosomes and Use Protein as a Nutrient. Cell.

[26] Han J.M., Jeong S.J., Park M.C., Kim G., Kwon N.H., Kim H.K., Ha S.H., Ryu S.H., Kim S. (2012). Leucyl-tRNA synthetase is an intracellular leucine sensor for the mTORC1-signaling pathway. Cell.

[27] Tsun Z.Y., Bar-Peled L., Chantranupong L., Zoncu R., Wang T., Kim C., Spooner E., Sabatini D.M. (2013). The folliculin tumor suppressor is a GAP for the RagC/D GTPases that signal amino acid levels to mTORC1. Mol. Cell.

[28] Kamada Y., Yoshino K., Kondo C., Kawamata T., Oshiro N., Yonezawa K., Ohsumi Y. (2010). Tor directly controls the Atg1 kinase complex to regulate autophagy. Mol. Cell Biol..

[29] Yuan H.X., Russell R.C., Guan K.L. (2013). Regulation of PIK3C3/VPS34 complexes by MTOR in nutrient stress-induced autophagy. Autophagy.

[30] Ma X., Zhang S., He L., Rong Y., Brier L.W., Sun Q., Liu R., Fan W., Chen S., Yue Z. (2017). MTORC1-mediated NRBF2 phosphorylation functions as a switch for the class III PtdIns3K and autophagy. Autophagy.

[31] Wong P.M., Feng Y., Wang J., Shi R., Jiang X. (2015). Regulation of autophagy by coordinated action of mTORC1 and protein phosphatase 2A. Nat. Commun..

[32] Wan W., You Z., Zhou L., Xu Y., Peng C., Zhou T., Yi C., Shi Y., Liu W. (2018). mTORC1-Regulated and HUWE1-Mediated WIPI2 Degradation Controls Autophagy Flux. Mol. Cell.

[33] Cheng X., Ma X., Zhu Q., Song D., Ding X., Li L., Jiang X., Wang X., Tian R., Su H. (2019). Pacer Is a Mediator of mTORC1 and GSK3-TIP60 Signaling in Regulation of Autophagosome Maturation and Lipid Metabolism. Mol. Cell.

[34] Kim Y.M., Jung C.H., Seo M., Kim E.K., Park J.M., Bae S.S., Kim D.H. (2015). mTORC1 phosphorylates UVRAG to negatively regulate autophagosome and endosome maturation. Mol. Cell.

[35] Martina J.A., Chen Y., Gucek M., Puertollano R. (2012). MTORC1 functions as a transcriptional regulator of autophagy by preventing nuclear transport of TFEB. Autophagy..

[36] Kim E., Goraksha-Hicks P., Li L., Neufeld T.P., Guan K.L. (2008). Regulation of TORC1 by Rag GTPases in nutrient response. Nat. Cell Biol..

[37] Sancak Y., Peterson T.R., Shaul Y.D., Lindquist R.A., Thoreen C.C., Bar-Peled L., Sabatini D.M. (2008). The Rag GTPases bind raptor and mediate amino acid signaling to mTORC1. Science.

[38] Dong J., Qiu H., Garcia-Barrio M., Anderson J., Hinnebusch A.G. (2000). Uncharged tRNA activates GCN2 by displacing the protein kinase moiety from a bipartite tRNA-binding domain. Mol. Cell.

[39] B’Chir W., Maurin A.C., Carraro V., Averous J., Jousse C., Muranishi Y., Parry L., Stepien G., Fafournoux P., Bruhat A. (2013). The eIF2α/ATF4 pathway is essential for stress-induced autophagy gene expression. Nucleic Acids Res..

[40] Seibenhener M.L., Babu J.R., Geetha T., Wong H.C., Krishna N.R., Wooten M.W. (2004). Sequestosome 1/p62 is a polyubiquitin chain binding protein involved in ubiquitin proteasome degradation. Mol. Cell Biol..

[41] Xia X.J., Gao Y.Y., Zhang J., Wang L., Zhao S., Che Y.Y., Ao C.J., Yang H.J., Wang J.Q., Lei L.C. (2016). Autophagy mediated by arginine depletion activation of the nutrient sensor GCN2 contributes to interferon-γ-induced malignant transformation of primary bovine mammary epithelial cells. Cell Death Discov..

[42] Jin H.R., Du C.H., Wang C.Z., Yuan C.S., Du W. (2019). Ginseng metabolite Protopanaxadiol induces Sestrin2 expression and AMPK activation through GCN2 and PERK. Cell Death Dis..

[43] Ye J., Palm W., Peng M., King B., Lindsten T., Li M.O., Koumenis C., Thompson C.B. (2015). GCN2 sustains mTORC1 suppression upon amino acid deprivation by inducing Sestrin2. Genes Dev..

[44] Wengrod J., Wang D., Weiss S., Zhong H., Osman I., Gardner L.B. (2015). Phosphorylation of eIF2α triggered by mTORC1 inhibition and PP6C activation is required for autophagy and is aberrant in PP6C-mutated melanoma. Sci. Signal..

[45] Tan P., Liu H., Zhao J., Gu X., Wei X., Zhang X., Ma N., Johnston L.J., Bai Y., Zhang W. (2021). Amino acids metabolism by rumen microorganisms: Nutrition and ecology strategies to reduce nitrogen emissions from the inside to the outside. Sci. Total Environ..

[46] Wolfson R.L., Chantranupong L., Wyant G.A., Gu X., Orozco J.M., Shen K., Condon K.J., Petri S., Kedir J., Scaria S.M. (2017). KICSTOR recruits GATOR1 to the lysosome and is necessary for nutrients to regulate mTORC1. Nature.

[47] Menon S., Dibble C.C., Talbott G., Hoxhaj G., Valvezan A.J., Takahashi H., Cantley L.C., Manning B.D. (2014). Spatial control of the TSC complex integrates insulin and nutrient regulation of mTORC1 at the lysosome. Cell.

[48] Morris S.M. (2016). Arginine Metabolism Revisited. J. Nutr..

[49] Saxton R.A., Chantranupong L., Knockenhauer K.E., Schwartz T.U., Sabatini D.M. (2016). Mechanism of arginine sensing by CASTOR1 upstream of mTORC1. Nature.

[50] Yao K., Yin Y.L., Chu W., Liu Z., Deng D., Li T., Huang R., Zhang J., Tan B., Wang W. (2008). Dietary arginine supplementation increases mTOR signaling activity in skeletal muscle of neonatal pigs. J. Nutr..

[51] Chantranupong L., Wolfson R.L., Orozco J.M., Saxton R.A., Scaria S.M., Bar-Peled L., Spooner E., Isasa M., Gygi S.P., Sabatini D.M. (2014). The Sestrins interact with GATOR2 to negatively regulate the amino-acid-sensing pathway upstream of mTORC1. Cell Rep..

[52] Jung J., Genau H.M., Behrends C. (2015). Amino Acid-Dependent mTORC1 Regulation by the Lysosomal Membrane Protein SLC38A9. Mol. Cell Biol..

[53] Rebsamen M., Pochini L., Stasyk T., de Araújo M.E., Galluccio M., Kandasamy R.K., Snijder B., Fauster A., Rudashevskaya E.L., Bruckner M. (2015). SLC38A9 is a component of the lysosomal amino acid sensing machinery that controls mTORC1. Nature.

[54] Wang S., Tsun Z.Y., Wolfson R.L., Shen K., Wyant G.A., Plovanich M.E., Yuan E.D., Jones T.D., Chantranupong L., Comb W. (2015). Metabolism. Lysosomal amino acid transporter SLC38A9 signals arginine sufficiency to mTORC1. Science.

[55] Jung J.W., Macalino S.J.Y., Cui M., Kim J.E., Kim H.J., Song D.G., Nam S.H., Kim S., Choi S., Lee J.W. (2019). Transmembrane 4 L Six Family Member 5 Senses Arginine for mTORC1 Signaling. Cell Metab..

[56] Kim J.S., Ro S.H., Kim M., Park H.W., Semple I.A., Park H., Cho U.S., Wang W., Guan K.L., Karin M. (2015). Sestrin2 inhibits mTORC1 through modulation of GATOR complexes. Sci. Rep..

[57] Baumann K. (2015). How mTORC1 senses leucine. Nat. Rev. Mol. Cell Biol..

[58] Wolfson R.L., Chantranupong L., Saxton R.A., Shen K., Scaria S.M., Cantor J.R., Sabatini D.M. (2016). Sestrin2 is a leucine sensor for the mTORC1 pathway. Science.

[59] Saxton R.A., Knockenhauer K.E., Wolfson R.L., Chantranupong L., Pacold M.E., Wang T., Schwartz T.U., Sabatini D.M. (2016). Structural basis for leucine sensing by the Sestrin2-mTORC1 pathway. Science.

[60] Hallett J.E.H., Manning B.D. (2016). CASTORing New Light on Amino Acid Sensing. Cell.

[61] Yoon M.S., Du G., Backer J.M., Frohman M.A., Chen J. (2011). Class III PI-3-kinase activates phospholipase D in an amino acid-sensing mTORC1 pathway. J. Cell Biol..

[62] Lee M., Kim J.H., Yoon I., Lee C., Fallahi Sichani M., Kang J.S., Kang J., Guo M., Lee K.Y., Han G. (2018). Coordination of the leucine-sensing Rag GTPase cycle by leucyl-tRNA synthetase in the mTORC1 signaling pathway. Proc. Natl. Acad. Sci. USA.

[63] Lorin S., Tol M.J., Bauvy C., Strijland A., Poüs C., Verhoeven A.J., Codogno P., Meijer A.J. (2013). Glutamate dehydrogenase contributes to leucine sensing in the regulation of autophagy. Autophagy.

[64] Jewell J.L., Kim Y.C., Russell R.C., Yu F.X., Park H.W., Plouffe S.W., Tagliabracci V.S., Guan K.L. (2015). Metabolism. Differential regulation of mTORC1 by leucine and glutamine. Science.

[65] Frigerio F., Karaca M., De Roo M., Mlynárik V., Skytt D.M., Carobbio S., Pajęcka K., Waagepetersen H.S., Gruetter R., Muller D. (2012). Deletion of glutamate dehydrogenase 1 (Glud1) in the central nervous system affects glutamate handling without altering synaptic transmission. J. Neurochem..

[66] Zhou Y., Ren J., Song T., Peng J., Wei H. (2016). Methionine Regulates mTORC1 via the T1R1/T1R3-PLCβ-Ca (2+)-ERK1/2 Signal Transduction Process in C2C12 Cells. Int. J. Mol. Sci..

[67] Yu M., Luo C., Huang X., Chen D., Li S., Qi H., Gao X. (2019). Amino acids stimulate glycyl-tRNA synthetase nuclear localization for mammalian target of rapamycin expression in bovine mammary epithelial cells. J. Cell Physiol..

[68] Longchamp A., Mirabella T., Arduini A., MacArthur M.R., Das A., Treviño-Villarreal J.H., Hine C., Ben-Sahra I., Knudsen N.H., Brace L.E. (2018). Amino Acid Restriction Triggers Angiogenesis via GCN2/ATF4 Regulation of VEGF and H(2)S Production. Cell.

[69] Konrad C., Wek R.C., Sullivan W.J. (2014). GCN2-like eIF2α kinase manages the amino acid starvation response in Toxoplasma gondii. Int. J. Parasitol..

[70] Rzymski T., Milani M., Pike L., Buffa F., Mellor H.R., Winchester L., Pires I., Hammond E., Ragoussis I., Harris A.L. (2010). Regulation of autophagy by ATF4 in response to severe hypoxia. Oncogene.

[71] García-Navas R., Munder M., Mollinedo F. (2012). Depletion of L-arginine induces autophagy as a cytoprotective response to endoplasmic reticulum stress in human T lymphocytes. Autophagy.

[72] Savaraj N., You M., Wu C., Wangpaichitr M., Kuo M.T., Feun L.G. (2010). Arginine deprivation, autophagy, apoptosis (AAA) for the treatment of melanoma. Curr. Mol. Med..

[73] Qiu F., Chen Y.R., Liu X., Chu C.Y., Shen L.J., Xu J., Gaur S., Forman H.J., Zhang H., Zheng S. (2014). Arginine starvation impairs mitochondrial respiratory function in ASS1-deficient breast cancer cells. Sci. Signal..

[74] Zhang X., Jin L., Tian Z., Wang J., Yang Y., Liu J., Chen Y., Hu C., Chen T., Zhao Y. (2019). Nitric oxide inhibits autophagy and promotes apoptosis in hepatocellular carcinoma. Cancer Sci..

[75] Stacchiotti A., Corsetti G. (2020). Natural Compounds and Autophagy: Allies Against Neurodegeneration. Front. Cell Dev. Biol..

[76] Sivangala Thandi R., Radhakrishnan R.K., Tripathi D., Paidipally P., Azad A.K., Schlesinger L.S., Samten B., Mulik S., Vankayalapati R. (2020). Ornithine-A urea cycle metabolite enhances autophagy and controls Mycobacterium tuberculosis infection. Nat. Commun..

[77] Yokota S.I., Ando M., Aoyama S., Nakamura K., Shibata S. (2016). Leucine restores murine hepatic triglyceride accumulation induced by a low-protein diet by suppressing autophagy and excessive endoplasmic reticulum stress. Amino Acids.

[78] Yan X., Sun Q., Ji J., Zhu Y., Liu Z., Zhong Q. (2012). Reconstitution of leucine-mediated autophagy via the mTORC1-Barkor pathway in vitro. Autophagy.

[79] Wu H., Wang F., Hu S., Yin C., Li X., Zhao S., Wang J., Yan X. (2012). MiR-20a and miR-106b negatively regulate autophagy induced by leucine deprivation via suppression of ULK1 expression in C2C12 myoblasts. Cell Signal..

[80] Sheen J.H., Zoncu R., Kim D., Sabatini D.M. (2011). Defective regulation of autophagy upon leucine deprivation reveals a targetable liability of human melanoma cells in vitro and in vivo. Cancer Cell.

[81] Dziedzic S.A., Caplan A.B. (2012). Autophagy proteins play cytoprotective and cytocidal roles in leucine starvation-induced cell death in Saccharomyces cerevisiae. Autophagy.

[82] Cruzat V., Macedo Rogero M., Noel Keane K., Curi R., Newsholme P. (2018). Glutamine: Metabolism and Immune Function, Supplementation and Clinical Translation. Nutrients.

[83] Tan H.W.S., Sim A.Y.L., Long Y.C. (2017). Glutamine metabolism regulates autophagy-dependent mTORC1 reactivation during amino acid starvation. Nat. Commun..

[84] Lin T.C., Chen Y.R., Kensicki E., Li A.Y., Kong M., Li Y., Mohney R.P., Shen H.M., Stiles B., Mizushima N. (2012). Autophagy: Resetting glutamine-dependent metabolism and oxygen consumption. Autophagy.

[85] Chen R., Zou Y., Mao D., Sun D., Gao G., Shi J., Liu X., Zhu C., Yang M., Ye W. (2014). The general amino acid control pathway regulates mTOR and autophagy during serum/glutamine starvation. J. Cell Biol..

[86] Sandri M. (2012). FOXOphagy path to inducing stress resistance and cell survival. Nat. Cell Biol..

[87] Berlicki Ł. (2008). Inhibitors of glutamine synthetase and their potential application in medicine. Mini Rev. Med. Chem..

[88] van der Vos K.E., Eliasson P., Proikas-Cezanne T., Vervoort S.J., van Boxtel R., Putker M., van Zutphen I.J., Mauthe M., Zellmer S., Pals C. (2012). Modulation of glutamine metabolism by the PI(3)K-PKB-FOXO network regulates autophagy. Nat. Cell Biol..

[89] Durán R.V., Oppliger W., Robitaille A.M., Heiserich L., Skendaj R., Gottlieb E., Hall M.N. (2012). Glutaminolysis activates Rag-mTORC1 signaling. Mol. Cell.

[90] Kim K.H., Lee M.S. (2014). Autophagy—a key player in cellular and body metabolism. Nat. Rev. Endocrinol..

[91] Eng C.H., Yu K., Lucas J., White E., Abraham R.T. (2010). Ammonia derived from glutaminolysis is a diffusible regulator of autophagy. Sci. Signal..

[92] Tang Y., Tan B., Xiong X., Li F., Ren W., Kong X., Qiu W., Hardwidge P.R., Yin Y. (2015). Methionine deficiency reduces autophagy and accelerates death in intestinal epithelial cells infected with enterotoxigenic Escherichia coli. Amino Acids.

[93] Ruckenstuhl C., Netzberger C., Entfellner I., Carmona-Gutierrez D., Kickenweiz T., Stekovic S., Gleixner C., Schmid C., Klug L., Sorgo A.G. (2014). Lifespan extension by methionine restriction requires autophagy-dependent vacuolar acidification. PLoS Genet..

[94] Pennington S.M., Klutho P.R., Xie L., Broadhurst K., Koval O.M., McCormick M.L., Spitz D.R., Grumbach I.M. (2018). Defective protein repair under methionine sulfoxide A deletion drives autophagy and ARE-dependent gene transcription. Redox Biol..

[95] Li R., Wei X., Jiang D.S. (2019). Protein methylation functions as the posttranslational modification switch to regulate autophagy. Cell Mol. Life Sci..

[96] Wu X., Tu B.P. (2011). Selective regulation of autophagy by the Iml1-Npr2-Npr3 complex in the absence of nitrogen starvation. Mol. Biol Cell.

[97] Neklesa T.K., Davis R.W. (2009). A genome-wide screen for regulators of TORC1 in response to amino acid starvation reveals a conserved Npr2/3 complex. PLoS Genet..

[98] Sutter B.M., Wu X., Laxman S., Tu B.P. (2013). Methionine inhibits autophagy and promotes growth by inducing the SAM-responsive methylation of PP2A. Cell.

[99] Murugan A.K. (2019). mTOR: Role in cancer, metastasis and drug resistance. Semin Cancer Biol..

[100] Kandasamy P., Gyimesi G., Kanai Y., Hediger M.A. (2018). Amino acid transporters revisited: New views in health and disease. Trends Biochem. Sci..

[101] Cheng C.T., Qi Y., Wang Y.C., Chi K.K., Chung Y., Ouyang C., Chen Y.R., Oh M.E., Sheng X., Tang Y. (2018). Arginine starvation kills tumor cells through aspartate exhaustion and mitochondrial dysfunction. Commun Biol..

[102] Strekalova E., Malin D., Good D.M., Cryns V.L. (2015). Methionine Deprivation Induces a Targetable Vulnerability in Triple-Negative Breast Cancer Cells by Enhancing TRAIL Receptor-2 Expression. Clin. Cancer Res..

[103] Liu H., Zhang W., Wang K., Wang X., Yin F., Li C., Wang C., Zhao B., Zhong C., Zhang J. (2015). Methionine and cystine double deprivation stress suppresses glioma proliferation via inducing ROS/autophagy. Toxicol Lett..

[104] Bernfeld E., Foster D.A. (2019). Glutamine as an Essential Amino Acid for KRas-Driven Cancer Cells. Trends Endocrinol. Metab..

[105] Cooke J.P., Singer A.H., Tsao P., Zera P., Rowan R.A., Billingham M.E. (1992). Antiatherogenic effects of L-arginine in the hypercholesterolemic rabbit. J. Clin. Investig..

[106] Khalaf D., Krüger M., Wehland M., Infanger M., Grimm D. (2019). The Effects of Oral l-Arginine and l-Citrulline Supplementation on Blood Pressure. Nutrients.

[107] van Loon L.J., Kruijshoop M., Menheere P.P., Wagenmakers A.J., Saris W.H., Keizer H.A. (2003). Amino acid ingestion strongly enhances insulin secretion in patients with long-term type 2 diabetes. Diabetes Care.

[108] Hu S., Han M., Rezaei A., Li D., Wu G., Ma X. (2017). L-Arginine Modulates Glucose and Lipid Metabolism in Obesity and Diabetes. Curr. Protein Pept. Sci..

[109] Liu H., Zhang J., Zhang S., Yang F., Thacker P.A., Zhang G., Qiao S., Ma X. (2014). Oral administration of Lactobacillus fermentum I5007 favors intestinal development and alters the intestinal microbiota in formula-fed piglets. J. Agric. Food Chem..

[110] Lei H., Yu B., Yang X., Liu Z., Huang Z., Mao X., Tian G., He J., Han G., Chen H. (2011). Inhibition of adipogenic differentiation by myostatin is alleviated by arginine supplementation in porcine-muscle-derived mesenchymal stem cells. Sci. China Life Sci..

[111] Chen X., Luo Y., Jia G., Liu G., Zhao H., Huang Z. (2017). The effect of arginine on the Wnt/β-catenin signaling pathway during porcine intramuscular preadipocyte differentiation. Food Funct..

[112] Miller R.A., Shi Y., Lu W., Pirman D.A., Jatkar A., Blatnik M., Wu H., Cárdenas C., Wan M., Foskett J.K. (2018). Targeting hepatic glutaminase activity to ameliorate hyperglycemia. Nat. Med..

[113] Ravindran R., Loebbermann J., Nakaya H.I., Khan N., Ma H., Gama L., Machiah D.K., Lawson B., Hakimpour P., Wang Y.C. (2016). The amino acid sensor GCN2 controls gut inflammation by inhibiting inflammasome activation. Nature.

[114] Yoon B.R., Oh Y.J., Kang S.W., Lee E.B., Lee W.W. (2018). Role of SLC7A5 in Metabolic Reprogramming of Human Monocyte/Macrophage Immune Responses. Front. Immunol..

[115] Sinclair L.V., Rolf J., Emslie E., Shi Y.B., Taylor P.M., Cantrell D.A. (2013). Control of amino-acid transport by antigen receptors coordinates the metabolic reprogramming essential for T cell differentiation. Nat. Immunol..

[116] Carr E.L., Kelman A., Wu G.S., Gopaul R., Senkevitch E., Aghvanyan A., Turay A.M., Frauwirth K.A. (2010). Glutamine uptake and metabolism are coordinately regulated by ERK/MAPK during T lymphocyte activation. J. Immunol..

[117] Kono M., Yoshida N., Maeda K., Tsokos G.C. (2018). Transcriptional factor ICER promotes glutaminolysis and the generation of Th17 cells. Proc. Natl. Acad. Sci. USA.

[118] Takahashi S., Saegusa J., Sendo S., Okano T., Akashi K., Irino Y., Morinobu A. (2017). Glutaminase 1 plays a key role in the cell growth of fibroblast-like synoviocytes in rheumatoid arthritis. Arthritis Res. Ther..

[119] Kim K., Lee S.G., Kegelman T.P., Su Z.Z., Das S.K., Dash R., Dasgupta S., Barral P.M., Hedvat M., Diaz P. (2011). Role of excitatory amino acid transporter-2 (EAAT2) and glutamate in neurodegeneration: Opportunities for developing novel therapeutics. J. Cell Physiol..

[120] Vernizzi L., Paiardi C., Licata G., Vitali T., Santarelli S., Raneli M., Manelli V., Rizzetto M., Gioria M., Pasini M.E. (2020). Glutamine Synthetase 1 Increases Autophagy Lysosomal Degradation of Mutant Huntingtin Aggregates in Neurons, Ameliorating Motility in a Drosophila Model for Huntington’s Disease. Cells.

[121] Michaud M., Xie X., Bravo-San Pedro J.M., Zitvogel L., White E., Kroemer G. (2014). An autophagy-dependent anticancer immune response determines the efficacy of melanoma chemotherapy. Oncoimmunology.

[122] Parodi M., Pedrazzi M., Cantoni C., Averna M., Patrone M., Cavaletto M., Spertino S., Pende D., Balsamo M., Pietra G. (2015). Natural Killer (NK)/melanoma cell interaction induces NK-mediated release of chemotactic High Mobility Group Box-1 (HMGB1) capable of amplifying NK cell recruitment. Oncoimmunology.

[123] Rosenfeldt M.T., O’Prey J., Morton J.P., Nixon C., MacKay G., Mrowinska A., Au A., Rai T.S., Zheng L., Ridgway R. (2013). p53 status determines the role of autophagy in pancreatic tumour development. Nature.

[124] Kim R.H., Coates J.M., Bowles T.L., McNerney G.P., Sutcliffe J., Jung J.U., Gandour-Edwards R., Chuang F.Y., Bold R.J., Kung H.J. (2009). Arginine deiminase as a novel therapy for prostate cancer induces autophagy and caspase-independent apoptosis. Cancer Res..

[125] Demontis F., Perrimon N. (2010). FOXO/4E-BP signaling in Drosophila muscles regulates organism-wide proteostasis during aging. Cell.

[126] Lipinski M.M., Zheng B., Lu T., Yan Z., Py B.F., Ng A., Xavier R.J., Li C., Yankner B.A., Scherzer C.R. (2010). Genome-wide analysis reveals mechanisms modulating autophagy in normal brain aging and in Alzheimer’s disease. Proc. Natl. Acad. Sci. USA.

[127] Leidal A.M., Levine B., Debnath J. (2018). Autophagy and the cell biology of age-related disease. Nat. Cell Biol..

[128] Madeo F., Pietrocola F., Eisenberg T., Kroemer G. (2014). Caloric restriction mimetics: Towards a molecular definition. Nat. Rev. Drug Discov..

[129] Abdellatif M., Sedej S., Carmona-Gutierrez D., Madeo F., Kroemer G. (2018). Autophagy in Cardiovascular Aging. Circ. Res..

[130] Bravo-San Pedro J.M., Kroemer G., Galluzzi L. (2017). Autophagy and Mitophagy in Cardiovascular Disease. Circ. Res..

[131] Kimball S.R., Jefferson L.S. (2001). Regulation of protein synthesis by branched-chain amino acids. Curr. Opin. Clin. Nutr. Metab. Care..

[132] Kimball S.R., Jefferson L.S. (2006). Signaling pathways and molecular mechanisms through which branched-chain amino acids mediate translational control of protein synthesis. J. Nutr..

[133] van Loon L.J. (2012). Leucine as a pharmaconutrient in health and disease. Curr. Opin. Clin. Nutr. Metab. Care..

[134] Borack M.S., Volpi E. (2016). Efficacy and Safety of Leucine Supplementation in the Elderly. J. Nutr..

[135] Zhang K., Zhang Y., Gu L., Lan M., Liu C., Wang M., Su Y., Ge M., Wang T., Yu Y. (2018). Islr regulates canonical Wnt signaling-mediated skeletal muscle regeneration by stabilizing Dishevelled-2 and preventing autophagy. Nat. Commun..

[136] Zheng C., Yao J., Guo L., Cao Y., Liang Z., Yang X., Cai C. (2019). Leucine-induced promotion of post-absorptive EAA utilization and hepatic gluconeogenesis contributes to protein synthesis in skeletal muscle of dairy calves. J. Anim. Physiol. Anim. Nutr..

[137] Xiong Y., Fru M.F., Yu Y., Montani J.P., Ming X.F., Yang Z. (2014). Long term exposure to L-arginine accelerates endothelial cell senescence through arginase-II and S6K1 signaling. Aging.

[138] Bárcena C., Quirós P.M., Durand S., Mayoral P., Rodríguez F., Caravia X.M., Mariño G., Garabaya C., Fernández-García M.T., Kroemer G. (2018). Methionine Restriction Extends Lifespan in Progeroid Mice and Alters Lipid and Bile Acid Metabolism. Cell Rep..

[139] Settembre C., De Cegli R., Mansueto G., Saha P.K., Vetrini F., Visvikis O., Huynh T., Carissimo A., Palmer D., Klisch T.J. (2013). TFEB controls cellular lipid metabolism through a starvation-induced autoregulatory loop. Nat. Cell Biol..

[140] Zhang Y., Goldman S., Baerga R., Zhao Y., Komatsu M., Jin S. (2009). Adipose-specific deletion of autophagy-related gene 7 (atg7) in mice reveals a role in adipogenesis. Proc. Natl. Acad. Sci. USA.

[141] Soussi H., Reggio S., Alili R., Prado C., Mutel S., Pini M., Rouault C., Clément K., Dugail I. (2015). DAPK2 downregulation associates with attenuated adipocyte autophagic clearance in human obesity. Diabetes.

[142] Zhang F., Zhao S., Yan W., Xia Y., Chen X., Wang W., Zhang J., Gao C., Peng C., Yan F. (2016). Branched Chain Amino Acids Cause Liver Injury in Obese/Diabetic Mice by Promoting Adipocyte Lipolysis and Inhibiting Hepatic Autophagy. EBioMedicine.

[143] Makowski L., Chaib M., Rathmell J.C. (2020). Immunometabolism: From basic mechanisms to translation. Immunol. Rev..

[144] Li W., Qu G., Choi S.C., Cornaby C., Titov A., Kanda N., Teng X., Wang H., Morel L. (2019). Targeting T Cell Activation and Lupus Autoimmune Phenotypes by Inhibiting Glucose Transporters. Front. Immunol..

[145] Fontana L., Cummings N.E., Arriola Apelo S.I., Neuman J.C., Kasza I., Schmidt B.A., Cava E., Spelta F., Tosti V., Syed F.A. (2016). Decreased Consumption of Branched-Chain Amino Acids Improves Metabolic Health. Cell Rep..

[146] Tsukishiro T., Shimizu Y., Higuchi K., Watanabe A. (2000). Effect of branched-chain amino acids on the composition and cytolytic activity of liver-associated lymphocytes in rats. J. Gastroenterol. Hepatol..

